# Letter to the Editor: Reply to “Population Screening for Cirrhosis” by Thiele et al, describing the Florida Healthy Liver Program

**DOI:** 10.1097/HC9.0000000000000713

**Published:** 2025-06-09

**Authors:** Kristen L. Ryland, Cynthia de la Garza-Ramos, Denise Harnois, Tushar Patel, Cyneetha Strong, George Whiddon, Christopher Mulrooney, Alma Littles, Beau Toskich

**Affiliations:** 1 Division of Hepatology and Liver Transplantation, Mayo Clinic, Jacksonville, Florida, USA; 2 Department of Interventional Radiology, Mayo Clinic, Jacksonville, Florida, USA; 3 Florida State University PrimaryHealth™, Tallahassee, Florida, USA; 4 Florida State University College of Medicine, Tallahassee, Florida, USA

To the editor,

We commend Thiele et al^1^ for their article “Population Screening for Cirrhosis,” which outlined screening strategies for liver fibrosis and cirrhosis and barriers to implementation.

Identifying liver disease should occur in primary care to allow for earlier detection and treatment of complications, such as HCC. For this reason, Mayo Clinic Florida and Florida State University (FSU) College of Medicine launched the Florida Healthy Liver Program. This partnership between a tertiary care center and a primary care academic center was created to increase access to screening technologies to improve the detection and prevention of liver disease and liver cancer.

The Florida Healthy Liver Program pilot explored the possibility of screening high-risk patients for liver fibrosis within primary care using elastography. A portable FibroScan (Echosens) was donated by Mayo Clinic Florida to FSU PrimaryHealth, a primary care clinic in a medically underserved section of Tallahassee, Florida. The clinicians received liver disease education from hepatologists and certified technical hands-on training. The clinicians identified high-risk patients, including those with type 2 diabetes, metabolic syndrome, obesity, sleep apnea, at-risk alcohol use, and hepatitis B or C infection, and incorporated elastography into their daily practice.

During the initial 6-month pilot, 55 adult and pediatric patients underwent FibroScan screening. Of these, 42% individuals demonstrated both fibrosis and steatosis, emphasizing the dire need for this screening outside of the subspecialty setting. Education on liver disease and counseling was provided to promote healthy lifestyle changes, and referrals were made to local gastroenterologists. A forthcoming manuscript will further describe our experience.

FSU PrimaryHealth staff and clinicians affirmed that elastography screening was both feasible in this setting and was valued. Most patients expressed a desire to adhere to recommended healthy lifestyle changes to improve their future results. The clinicians describe using the FibroScan device as a “blood pressure cuff” for the liver and find it indispensable to their practice.

Challenges included an initial lack of staff availability for add-on scans and securing reliable patient transportation for subsequent appointments. Rarely, obtaining accurate elastography readings took several iterations, resulting in a slightly longer scan time.

The FSU PrimaryHealth practice will care for all current and future patients who screen positive for liver disease as risk factor modification extends beyond medical therapy and requires supporting nutritional, behavioral, socioeconomic challenges, and extrahepatic comorbidities. As such, the Florida Healthy Liver program strongly feels that primary care should be the “captain of the ship” in the early detection of this alarming threat to patients in Florida.

The Florida Healthy Liver Program plans to scale the pilot program to 6 other clinics. The program will further define the role and benefits of primary care liver disease screening, to include treatment of earlier stage disease with gastroenterologists and promote multidisciplinary management. We are confident this program will prove efficacious in broader population screening for liver fibrosis in primary care and plan to expand its scope by further engaging federal, industry, and legislative support to protect the health of our communities.
